# Intraductal carcinoma of the prostate: a critical re-appraisal

**DOI:** 10.1007/s00428-019-02544-6

**Published:** 2019-03-01

**Authors:** Murali Varma, Brett Delahunt, Lars Egevad, Hemamali Samaratunga, Glen Kristiansen

**Affiliations:** 1 Division of Cancer & Genetics, Cardiff University School of Medicine, Cardiff, UK; 2 Department of Pathology and Molecular Medicine, Wellington School of Medicine and Health Sciences, University of Otago Wellington, Wellington, New Zealand; 3 Department of Oncology-Pathology, Karolinska Institutet, Stockholm, Sweden; 4 Aquesta Uropathology, University of Queensland, Brisbane, Queensland Australia; 5 Institute of Pathology, University Hospital Bonn, Bonn, Germany

**Keywords:** Prostate cancer, Intraductal carcinoma of prostate gland, Ductal adenocarcinoma, Critical review

## Abstract

Intraductal carcinoma of the prostate gland (IDCP), which is now categorised as a distinct entity by WHO 2016, includes two biologically distinct diseases. IDCP associated with invasive carcinoma (IDCP-inv) generally represents a growth pattern of invasive prostatic adenocarcinoma while the rarely encountered pure IDCP is a precursor of prostate cancer. This review highlights issues that require further discussion and clarification. The diagnostic criterion “nuclear size at least 6 times normal” is ambiguous as “size” could refer to either nuclear area or diameter. If area, then this criterion could be re-defined as nuclear diameter at least three times normal as it is difficult to visually compare area of nuclei. It is also unclear whether IDCP could also include tumours with ductal morphology. There is no consensus whether pure IDCP in needle biopsies should be managed with re-biopsy or radical therapy. A pragmatic approach would be to recommend radical therapy only for extensive pure IDCP that is morphologically unequivocal for high-grade prostate cancer. Active surveillance is not appropriate when low-grade invasive cancer is associated with IDCP, as such patients usually have unsampled high-grade prostatic adenocarcinoma. It is generally recommended that IDCP component of IDCP-inv should be included in tumour extent but not grade. However, there are good arguments in favour of grading IDCP associated with invasive cancer. All historical as well as contemporary Gleason outcome data are based on morphology and would have included an associated IDCP component in the tumour grade. WHO 2016 recommends that IDCP should not be graded, but it is unclear whether this applies to both pure IDCP and IDCP-inv.

## Introduction

Intraductal carcinoma of the prostate gland (IDCP) is characterised by a lumen-spanning proliferation of atypical prostatic epithelium within expanded pre-existing prostatic ducts, with, at least, a partially preserved basal cell layer. The earliest description of IDCP, to our knowledge, was included in a systematic autopsy study published in 1938 by EP Gaynor [1]. It was, however, not until 1985 that IDCP was described as a distinct entity by Kovi et al. [2]. In this study, they demonstrated IDCP in 48% of 139 adenocarcinomas of the prostate in a series consisting mainly of transurethral resection (TUR) specimens. McNeal et al. provided a more detailed description of IDCP in 1986 and established its association with aggressive prostate cancer [3] and in 1996 described the key morphological criteria for the diagnosis of IDCP [4]. Guo and Epstein [5] refined these criteria to identify IDCP in needle biopsies and their criteria are currently those most frequently used to identify IDCP in all types of prostate specimens. IDCP was formalised as a biologically distinct entity in the 2016 edition of the World Health Organization (WHO) Classification of Tumours of the Prostate Gland [6].

In the past decade, there has been considerable interest in IDCP and several reviews of this entity have been published [7–10]. However, there are some significant issues relating to the diagnosis and reporting of IDCP that merit more detailed discussion. In this review, we focus on these more controversial issues, highlight areas that require further discussion and suggest some potential solutions.

## Nature of IDCP

Until recently, IDCP was an issue largely discussed by academic uropathologists. However, with the increasing awareness and reporting of this entity by practicing pathologists, there is a greater potential for misunderstanding its nature.

Since the publication of the seminal study of Kovi et al. [2], IDCP was considered to represent a growth pattern of invasive prostatic adenocarcinoma showing aggressive infiltration and expansion of pre-existing benign prostatic ducts (IDCP-inv). However, in 2010, Robinson and Epstein studied 21 specimens from radical prostatectomies following a diagnosis of pure IDCP in needle biopsies and described two cases in which the completely submitted prostate gland showed only IDCP with no co-existing component of invasive carcinoma identified [11]. Similarly, in 14 (2%) of 901 radical prostatectomy specimens studied by Miyai et al., IDCP was found to be spatially separate from invasive carcinoma and interpreted as “precursor-like IDCP” [12]. Thus, while IDCP generally represents a growth pattern of invasive prostatic adenocarcinoma, it may occasionally represent a precursor of prostatic adenocarcinoma. It is important to appreciate that the morphological entity “IDCP” includes two biologically distinct diseases, as this has significant implications for its diagnosis and reporting.

In prostate needle biopsies, IDCP is usually associated with an overtly invasive component of prostate carcinoma. The less common scenario where it is unaccompanied by invasive cancer has been referred to as pure or isolated IDCP. However, most instances of pure IDCP in prostate needle biopsy represent IDCP-inv with an unsampled invasive component.

Some pathologists and clinicians confuse IDCP with ductal carcinoma of the prostate. The term *ductal* in IDCP refers to the location of the tumour within large ducts, while *ductal* in ductal adenocarcinoma refers to the tumour cell phenotype, as ductal tumours are defined by their distinctive cytology.

It is unclear whether the term IDCP should be restricted to tumours showing an acinar phenotype or could also include tumours with ductal morphology. Papillary tumours with a prostatic ductal cellular morphology may have preserved basal cells, and such tumours have been variably classified as ductal adenocarcinoma, non-invasive ductal adenocarcinoma or IDCP [13–15]. In a recent survey of European pathologists, 39% of respondents noted that they would use the term IDCP only for acinar proliferations, while 58% would include tumours with ductal morphology (Unpublished Observation). This issue would be particularly important if IDCP is managed by re-biopsy rather than radical therapy. The terminology for non-invasive tumours of ductal phenotype needs to be standardised.

## Diagnosis of IDCP

The diagnosis of IDCP is generally made using the morphological criteria described by Guo and Epstein, which were recommended by the WHO in 2016 [5, 6]. It must be appreciated that Guo and Epstein set out criteria to identify pure IDCP in prostate needle biopsies, which would then be managed with radical therapy, even in the absence of a co-existing invasive component. Hence, these criteria were designed to include only cases in which the possibility of high-grade prostatic intraepithelial neoplasia (HGPIN) could be definitively excluded. The bar was therefore set very high as this definition of IDCP would exclude cases of IDCP in which the morphology overlaps with that of HGPIN.

It has recently been proposed that the spectrum of IDCP should be expanded by designating some atypical intraductal proliferations, which fall short morphologically of “classical” IDCP according to Guo and Epstein criteria, as low-grade IDCP (LGIDCP) [16]. Further, it has been recommended that this particularly applies if on immunostaining the tumours are ERG positive and PTEN negative, which is the typical immunoprofile of IDCP. These authors suggest that such proliferations should be managed by prompt re-biopsy rather than the radical therapy recommended by some experts for classical IDCP. Given the current state of uncertainty regarding the diagnosis and management of IDCP, the introduction of a new category of LGIDCP could cause significant confusion among pathologists and clinicians. Moreover, expansion of the spectrum of IDCP risks over-treatment with the potential for radical therapy for patients with low grade disease [17]. We prefer the more descriptive term “atypical proliferation suspicious for intraductal carcinoma” (ASID) for glandular proliferations that are morphologically indeterminate between HGPIN and IDCP [18]. Unlike LGIDCP, ASID should not be considered a diagnostic entity but merely an indication of diagnostic uncertainty analogous to ASAP (atypical small acinar proliferation) and PINATYP (HGPIN associated with atypical small acini suspicious for invasive cancer).

The reporting of ASID/LGIDCP morphology may be particularly important when encountered in association with low-grade invasive carcinoma as this could have management implications as detailed below.

Recent studies have shown significant inter-observer variation in the diagnosis of IDCP. Iczkowski et al. circulated photomicrographs to 39 uropathologists and reported only 43% agreement with the original diagnosis of IDCP [19]. Varma et al. surveyed 23 expert uropathologists and found significant variation in the diagnostic criteria and rules used to report IDCP [20]. With more widespread recognition of IDCP, through its acceptance as a novel entity by the WHO [6], there is a danger of an even greater degree of confusion and variation in the diagnosis and reporting of IDCP among non-specialist pathologists.

While the apparent lack of inter-observer reproducibility in the diagnosis of IDCP may be due to the inevitable variation in the interpretation of borderline morphology, it may also be due to an entirely avoidable variation in the interpretation of the definitions of diagnostic criteria. In view of this, diagnostic criteria for IDCP should be unambiguous to ensure consistent diagnosis and reporting.

Guo and Epstein proposed that solid and dense cribriform growth patterns are diagnostic of IDCP, while a diagnosis of IDCP would be rendered in loose cribriform and micropapillary proliferations only if there was marked nuclear enlargement or non-focal comedonecrosis. It is now clear that the definitions of “marked nuclear enlargement” and “dense” cribriform proliferation need further clarification.

**Fig. 1:**
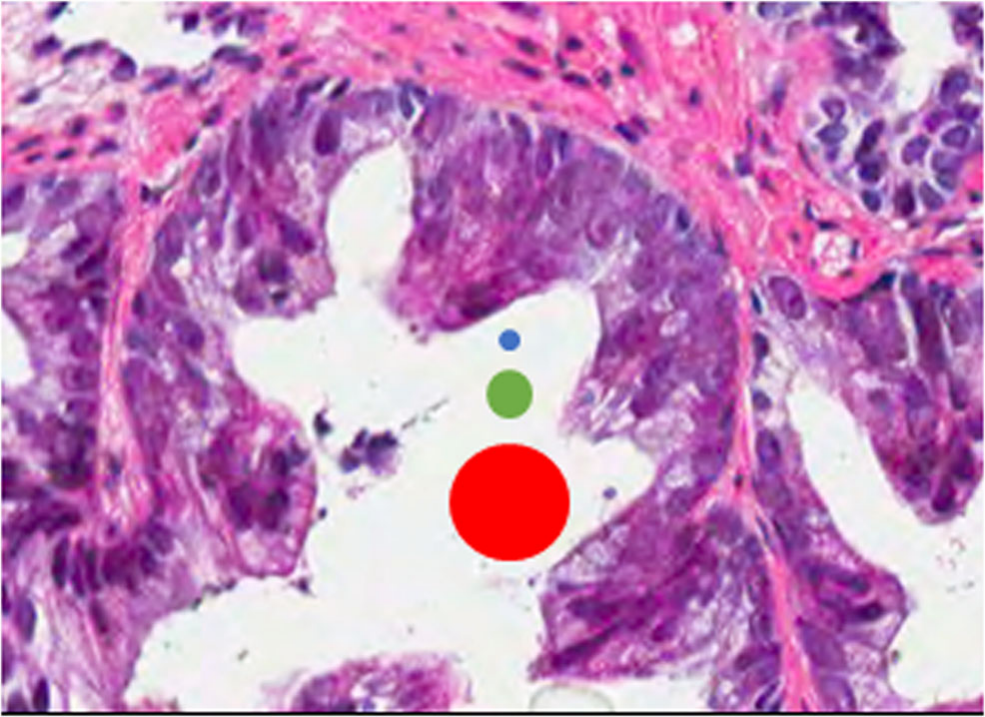
This case could meet “> six times normal” nuclear size criterion for intraductal carcinoma of the prostate if size is defined as nuclear area but not if defined as nuclear diameter (blue dot: size of normal nucleus, green dot: size six times normal area and red dot: size six times normal diameter)

Marked nuclear enlargement has been defined as a “nuclear size at least six times normal”, which has been has been variably interpreted as “size” may apply to nuclear diameter, radius or area. In a recent survey, 74% of expert uropathologists defined size by nuclear area and 21% by nuclear diameter [20]. A six times increase in nuclear diameter would be equivalent to a 36× increase in nuclear area, which would be very rarely encountered in clinical practice (Fig. 1). The nuclear size criterion was proposed by Guo and Epstein to improve reproducibility in the diagnosis of IDCP in the absence of solid growth pattern, dense cribriform growth pattern or comedonecrosis. However, inconsistent interpretation of this definition could lead to marked variation as some pathologists would require marked nuclear enlargement (greater than six times normal area) while others would require bizarre nuclei (greater than six times normal diameter). Defining size based on nuclear area would be problematic in routine practice as it is difficult to visually compare the area of nuclei. Hence, if this interpretation of nuclear size criterion is appropriate, then it could be re-defined as nuclear diameter at least three times normal (approximately equivalent to a nuclear area of at least six times normal) to avoid ambiguity. Moreover, since normal prostatic secretory cell nuclei can vary significantly, nuclear size could be defined in relation to that of lymphocytes or red blood cells.

The nuclear enlargement cut-off is arbitrary as there were no studies comparing outcome of various nuclear sizes so a visual estimate of nuclear size is sufficient. It may even be appropriate to redefine this criterion more simply as “severe nuclear enlargement” with publication of microphotographs to illustrate the minimum degree of nuclear enlargement required for a diagnosis of IDCP in this setting.

The dense cribriform pattern criterion was originally defined as foci in which “solid areas predominated over luminal spaces”, which would logically be interpreted as indicating proliferations that would consist of > 50% epithelium [5]. However, in a recent review paper, Wobker and Epstein re-defined dense cribriform pattern as “>70% epithelium as opposed to lumens” [9]. This raising of the bar for diagnosis of IDCP is prudent as it would reduce the risk of over-diagnosis of IDCP. This change does, however, need to be sufficiently emphasised to ensure that it does not lead to further variation in the diagnosis of IDCP, due to the existence of conflicting criteria.

“Non-focal comedonecrosis” is another criterion described for the diagnosis of IDCP in foci lacking solid or dense cribriform growth patterns [5]. However, true focality of comedonecrosis can be difficult to establish due to the intrinsic sampling error of needle biopsies. Comedonecrosis can be distinguished from intraluminal secretions by the presence of nuclear material and ragged luminal surface due to cellular necrosis. Most foci of morphologically comedonecrosis Gleason pattern 5 prostate cancer probably represent IDCP as discussed in the section on grading issues.

## Differential diagnosis

**Fig. 2:**
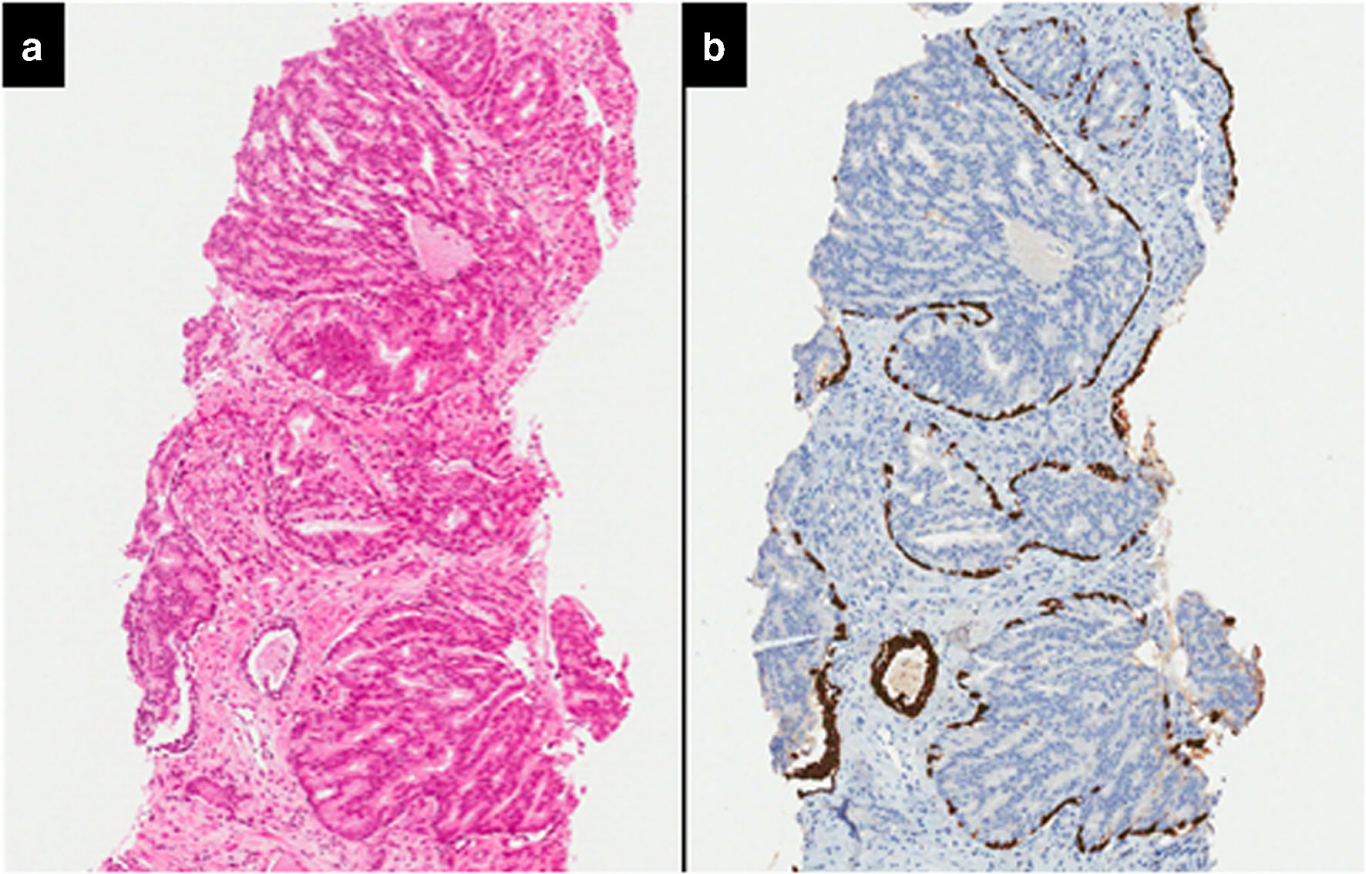
Intraductal carcinoma of the prostate with an infiltrative growth pattern may be morphologically difficult to distinguish from invasive cancer. One focus shows comedonecrosis morphologically suggesting Gleason pattern 5 invasive carcinoma (**a** haematoxylin and eosin, **b** CK5/6)

**Fig. 3:**
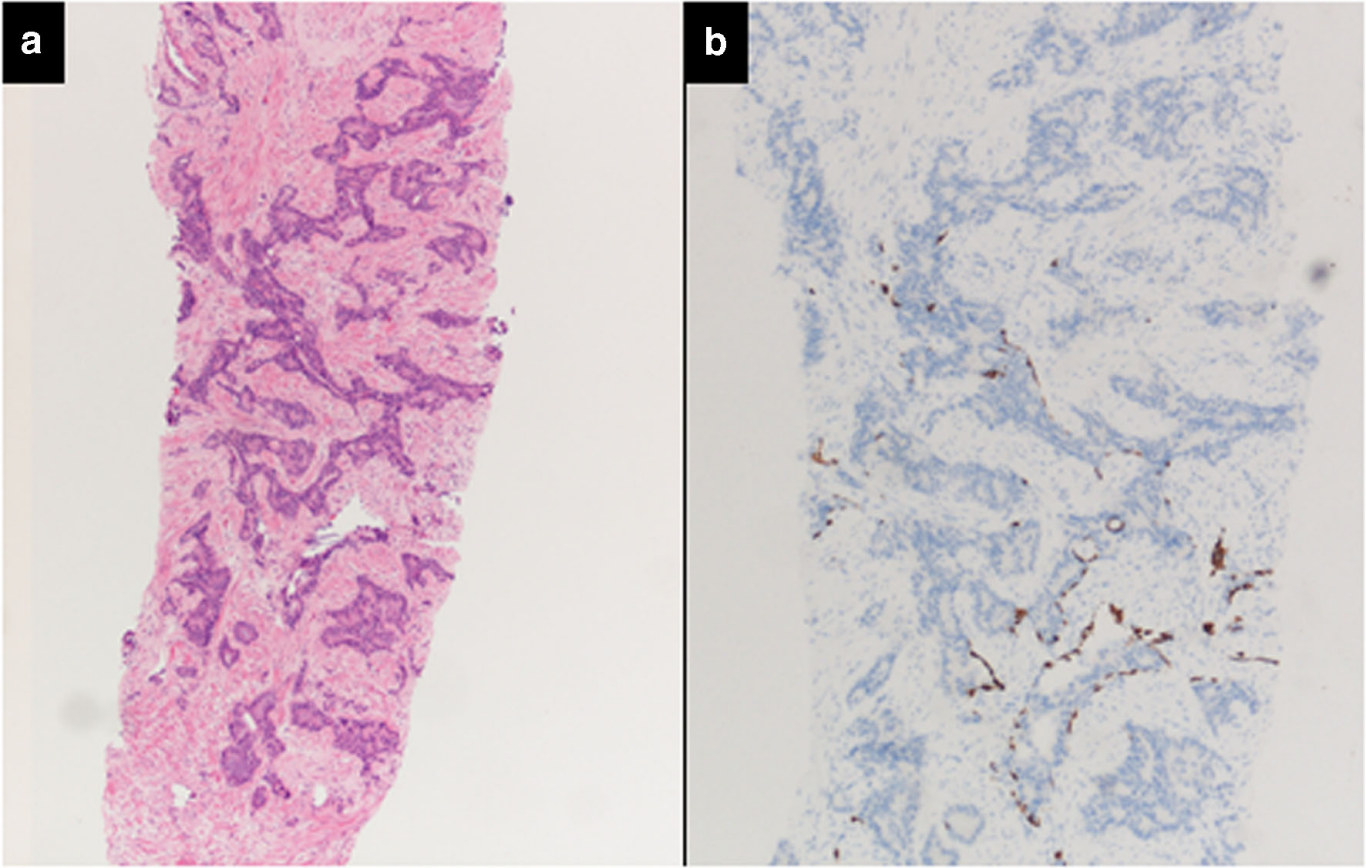
Intraductal carcinoma of the prostate with very patchy basal cells identified by immunohistochemistry. At least some of the glands lacking basal cell immunoreactivity represent intraductal rather than invasive carcinoma (**a** haematoxylin and eosin, **b** CK 5/6)

Although diagnostic criteria for IDCP were primarily designed to distinguish IDCP from HGPIN, the other major and difficult differential diagnosis is invasive prostate cancer. The accurate distinction of cribriform/comedonecrosis patterns of IDCP from cribriform/comedonecrosis invasive prostate cancer is often not possible without the aid of basal cell marker immunohistochemistry (Fig. 2). Hence, some studies analyse IDCP and invasive cancer with cribriform pattern together [21]. Basal cell marker immunoreactivity is often patchy in IDCP and as a consequence, even immunohistochemistry may not be conclusive in differentiating IDCP from invasive cancer. While the identification of basal cells would support a diagnosis of IDCP, the absence of basal cell marker immunoreactivity does not exclude the possibility of the suspect glands representing IDCP with absence of basal cells in the examined plane of section (Fig. 3). Other differential diagnoses include clear cell cribriform hyperplasia, ductal adenocarcinoma, PIN-like ductal carcinoma and urothelial carcinoma and the differentiating features of these lesions these have been extensively covered in recent reviews [7–10].

## Molecular pathology

Early studies on IDCP focussed on features such as proliferation or loss of heterozygosity (LOH) of common tumour suppressor genes. Cohen et al. analysed Ki-67 fractions in IDCP and described proliferation rates that were comparable to adjacent invasive carcinoma [22]. In a further study of this group analysing a set of 12 loci commonly altered in prostate cancer, LOH was found in 60% of IDCP [23]. In comparison, Gleason pattern 3 carcinoma showed no LOH, whereas Gleason pattern 4 tumours had 29% of LOH, indicating that IDCP might be even more disturbed that invasive carcinoma. Bettendorf et al. used PCR to demonstrate particularly high rates of LOH in *PTEN* (45%), *TP53* (60%) and *RB* (81%) [24]. However, these studies were conducted before the publication of the WHO 2016 definition of IDCP and hence the applied definitions of IDCP may vary.

The prevalence of *ETS* gene rearrangements in high grade PIN, invasive and intraductal carcinoma was analysed by Han et al., and they found that HGPIN in their cohort lacked *ERG* gene rearrangement, whereas it was present in 75% of IDCP, matching the positivity of adjacent invasive glands [25]. This was confirmed by Lotan et al. who studied immunohistochemical PTEN expression in prostatic tissues and proposed cytoplasmic PTEN loss as a helpful diagnostic criterion for the molecular separation of HGPIN (100% PTEN positive) from IDCP (only 16% PTEN positivity) [26]. Schneider and Osunkoya demonstrated that ERG immunoreactivity was comparable in ERG positive IDCP cases and adjacent invasive adjacent carcinoma, endorsing the assumption that intraductal carcinoma of the prostate probably represents colonization of benign glands by adjacent pre-existing conventional prostatic adenocarcinoma [27]. A detailed analysis of ERG and PTEN in HGPIN, invasive carcinoma and IDCP by Haffner et al. provided further evidence that invasive adenocarcinoma can morphologically mimic HGPIN through retrograde colonization of benign glands [28]. More recently, Tolkach et al. proposed a re-think of our definitions and concepts of “HGPIN” that is spatially associated with invasive carcinoma, as this may represent a post-invasive re-entry lesion and not, as assumed so far, a precursor lesion [29]. Atypical intraductal cribriform proliferations (AIP) that fall short of the criteria of IDCP were examined by Hickman et al., who found similar ERG and PTEN expression patterns in AIP and IDCP, which, as they suggest, might imply a similar clinical relevance [30].

Apart from PTEN and ERG, no other molecular markers are, as yet, established as diagnostic markers of IDCP. IDCP has been associated with *BRCA2* defects in familial prostate cancer, as it was shown that intraductal growth was significantly more prevalent in xenografts from BRCA2-mutated cases than in sporadic cases [31]. However, a comparison of Gleason score matched primary cases of familial vs. sporadic cases is to our knowledge still lacking. Lindberg et al. conducted a detailed comparison of copy-number variations (CNV) in nodal metastasis of a case of prostate cancer with those in 34 different foci of the corresponding primary tumour [32]. Surprisingly, the closest molecular semblance to lymph node metastasis was found in the focus of IDCP. As a purely intraductal process would be unable to metastasise, it is likely that a corresponding invasive focus was not sampled for the CNV analysis. However, these findings are consistent with intraductal growth being a hallmark of more aggressive tumours and or even that the intraductal component of a tumour is enriched for tumour cells with a particularly aggressive behaviour. The role of the inevitable intraductal hypoxia that might promote further tumour progression in IDCP has not yet been clarified, even though hypoxia has long been recognised as an accelerator of tumour dedifferentiation and progression [33, 34]. The general association of IDCP, genomic instability and hypoxia was recently analysed in an impressive multicenter study that also confirmed the prognostic value of IDCP or glands with cribriform growth (CA+) [35]. Furthermore, they demonstrated that IDCP/CA+ prostate cancers had increased hypoxic tumour subpopulations when compared with IDCP/CA– prostate cancers and that they also exhibited increased percentages of genomic alterations. Finally, they found the long non coding RNA *SChLAP1* at threefold higher levels in IDCP/CA+ cases, which warrants further study to clarify its biological or even diagnostic role. The association of IDCP/CA+ with genomic instability was also confirmed in a clever re-analysis of publically available TCGA data, which also showed higher rates of point mutations in *TP53*, *SPOP* and *FOXA1* in these cases [36].

## Frequency of IDCP

The reported incidence of IDCP in needle biopsies and radical prostatectomy specimens varies widely depending on the patient cohort studied, as IDCP is more commonly seen in association with high-grade, high-stage invasive prostate cancer. In one large series, Watts et al. found IDCP in 2.8% of 1176 consecutive prostate biopsies, including pure IDCP in 0.26% [37].

The three proven cases of pure IDCP without an associated invasive tumour component in the prostate gland were in radical prostatectomy specimens from series where radical therapy was offered to patients identified as having pure IDCP in needle biopsies [11, 38]. The true incidence of pure IDCP is unknown although the comprehensive examination of cystoprostatectomy specimens could provide useful information. These specimens were used in the study of Siadat et al. reported in 2015 [39]; however, they analysed only specimens with, at least, Gleason score 7 tumours, while there was only partial sampling of the specimens submitted for histological examination in the series examined by Morais et al. [40].

Before rendering a diagnosis of pure IDCP in a needle biopsy set, a diligent search for invasive carcinoma is required. If pure IDCP is suspected, the examination of deeper levels must be considered in order to determine the potential presence of an albeit limited component of invasive tumour.

## Clinical significance

Several studies have demonstrated the clinical significance of IDCP, both in needle biopsies and in radical prostatectomy specimens.

The presence of an IDCP component within a prostate cancer diagnosed on needle biopsy has been shown to correlate with increased risk of tumour recurrence and reduced survival [41]. IDCP in radical prostatectomy specimens has also been correlated with high-stage disease and shown to be a predictor of post-surgical biochemical recurrence [42].

IDCP-inv in needle biopsies is generally associated with extensive high-grade prostate cancer in the same specimen, as well as in the corresponding radical prostatectomy [7–10]. In view of this, it is not surprising that some studies have suggested that IDCP-inv is associated with an increased rate of biochemical recurrence and metastasis after radiotherapy, as well as resistance to androgen suppression and chemotherapy [43].

Occasionally, IDCP-inv in a needle biopsy may be associated with low-grade invasive prostate cancer. Khani and Epstein described the outcome of 62 patients in whom the needle biopsy showed IDCP and Gleason score 3 + 3 = 6 adenocarcinoma [44]. Six percent of these men had metastatic disease at presentation. Of the 45 men who received radical therapy, 20% developed disease progression within 3 years, 13% ultimately developed metastatic disease and 7% died of disease. Thus, the clinical and pathological outcomes of Gleason score 3 + 3 = 6 invasive cancer associated with an IDCP component in biopsies are clearly very different from that of usual Gleason score 3 + 3 = 6 prostate cancer without associated IDCP.

Pure IDCP in needle biopsies generally represents IDCP-inv with an unsampled invasive component, and its distinction from HGPIN is particularly important in contemporary practice, as current guidelines do not recommend routine re-biopsy for the latter, particularly when focal.

## Management implications

The management of patients with pure IDCP in needle biopsies is controversial. Some experts recommend radical therapy even in the absence of an associated invasive component as such patients often have high-grade, locally advanced or metastatic prostatic cancer [5, 15]. On the contrary, other experts favour re-biopsy as some patients may have only pure IDCP in the subsequent radical prostatectomy specimen [45].

Men with pure IDCP should, at least, undergo prompt multiparametric MRI examination and re-biopsy. Due to the association between IDCP and high-volume invasive prostate cancer, re-biopsy is likely to be positive. If; however, the re-biopsy shows no invasive malignancy, then there is uncertainty as to how the patient should be managed. Unlike low volume Gleason 3 + 3 = 6 invasive prostate cancer, delay in the commencement of therapy following a diagnosis of pure IDCP in a needle biopsy could have serious consequences if there is occult high-grade cancer elsewhere in the prostate gland.

A pragmatic approach would be to recommend radical therapy for extensive pure IDCP that is morphologically unequivocal for high-grade prostate cancer and re-biopsy for IDCP with features that are morphologically equivocal for invasive carcinoma [8]. Adoption of such a strategy could reduce overuse of immunohistochemistry to exclude the possibility of IDCP in cases where the morphology is of high-grade invasive cancer (Figs. 2 and 3).

In contrast to the controversy surrounding the management of pure IDCP, there is general consensus that active surveillance is not an appropriate option when low-grade invasive cancer is associated with IDCP, as such patients generally have unsampled high-grade prostatic malignancy [44]. This scenario, however, is rare, and there is a need for further studies to determine whether active surveillance could be considered for men with negative MRI and only focal IDCP associated with low-grade invasive cancer. Similarly, it is unclear whether men on an active surveillance program with stable PSA levels and radiological findings should have radical therapy, if a routine re-biopsy shows focal IDCP associated with low-grade invasive cancer.

**Fig. 4:**
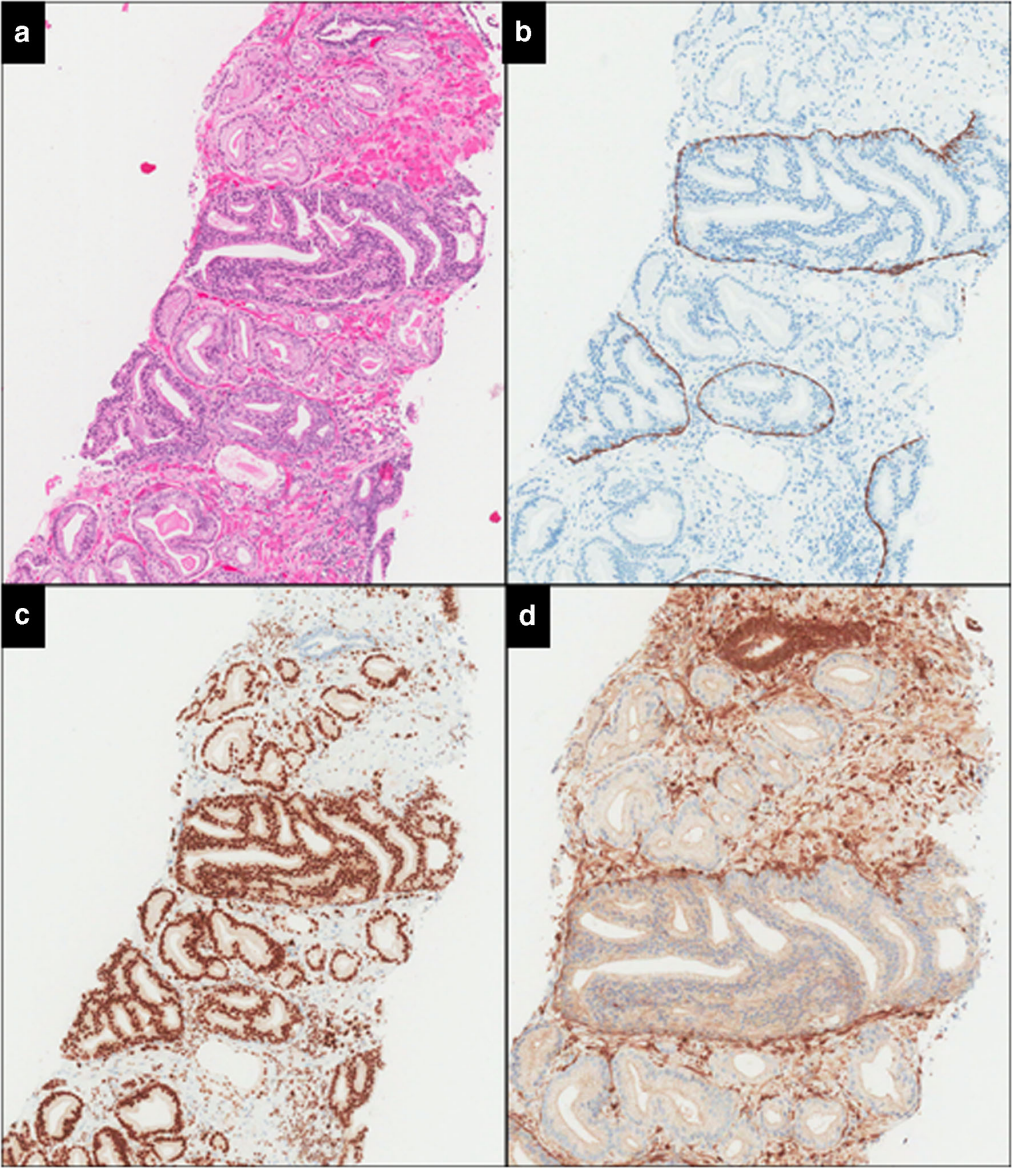
ISUP grade 1 invasive cancer associated with a loose cribriform proliferation, which is morphologically Gleason pattern 4 but shows a prominent basal cell layer and is ERG positive and PTEN negative. However, the cribriform proliferation lacks marked nuclear atypia or comedonecrosis to warrant a diagnosis of intraductal carcinoma and is interpreted as atypical proliferation suspicious for intraductal carcinoma (ASID) (**a** haematoxylin and eosin, **b** CK5/6, **c** ERG, **d** PTEN)

Another clinical dilemma would be low-grade invasive carcinoma associated with LGIDCP/ASID. This is particularly so when the latter has the morphology of cribriform (Gleason pattern 4) invasive carcinoma with basal cells identified on immunohistochemical staining, but lacking the dense cribriform architecture, marked pleomorphism or comedonecrosis, which would warrant a diagnosis of IDCP (Fig. 4). It is generally recognised that cribriform invasive carcinoma cannot be reliably distinguished from cribriform IDCP without immunohistochemistry (Figs. 2 and 3), and several studies suggest that the prognostic significance of cribriform carcinoma diagnosed by morphology alone is similar to that of IDCP [21, 45, 46]. Hence, radical therapy should be considered for low-grade invasive carcinoma associated with LGIDCP/ASID with morphology of cribriform (Gleason pattern 4) invasive carcinoma.

## Reporting of IDCP

### Tumour extent

Most experts recommend that an associated IDCP component be included when assessing tumour extent in biopsies with invasive prostate cancer [8, 20]. Arguments in favour of including IDCP in assessment of tumour extent include the difficulty in distinguishing IDCP and invasive components, the adverse prognostic significance of IDCP and that in most cases, this represents invasive carcinoma extending into benign ducts. A counter argument is that IDCP is not actually invasive in prostatic stroma and hence more comparable with vascular invasion, which is not included in tumour size estimation in other sites. We recommend that it must be clearly indicated in the report if the reported tumour extent has been significantly influenced by the presence of IDCP.

### Tumour grade

The appropriateness of Gleason grading of IDCP, particularly in biopsy material, is controversial. The International Society of Urological Pathologists (ISUP) consensus conference on prostate cancer grading, held in 2014, recommended that IDCP should not be graded and this was subsequently endorsed by the WHO in 2016 [6, 47].

As discussed earlier, IDCP includes two biologically distinct diseases that need to be considered separately. Pure IDCP is a precursor lesion analogous to HGPIN, while IDCP-inv generally represents a growth pattern of aggressive invasive carcinoma. Thus, having a single rule for reporting, all IDCP would be akin to uniform reporting guidelines for HGPIN and invasive carcinoma. This clearly would be inappropriate as HGPIN is not graded, while it is standard practice to report the Gleason score in most cases of invasive prostate cancer.

Unfortunately, grading based upon these two scenarios (pure IDCP and IDCP-inv) was not separately discussed at the 2014 ISUP consensus conference and is not mentioned in the 2014 ISUP grading classification for prostate cancer [47].

The main argument against grading IDCP is that tumour grading is designed and validated only for invasive carcinoma, while IDCP may represent a precursor lesion. Although most cases of pure IDCP in prostate needle biopsies represent IDCP-inv with an unsampled invasive component, it would be prudent not to grade pure IDCP as some cases appear to represent an aggressive precursor lesion rather than invasive prostate cancer. Moreover, there is no consensus regarding the appropriateness of radical therapy for pure IDCP and reporting a Gleason score in such cases may lead to urologists interpreting and treating it as invasive cancer. However, Gleason grading of IDCP-inv does merit more detailed discussion as there are several arguments in favour of including the IDCP component when grading IDCP-inv [48].

An IDCP component in IDCP-inv almost always represents a growth pattern of aggressive invasive carcinoma rather than an associated precursor lesion. Hence, one would generally be grading invasive tumour if this component is included in the Gleason score. The strongest argument favouring inclusion of IDCP component in Gleason score is that all historical as well as contemporary Gleason outcome data are based on morphology and would have included an associated IDCP component in the tumour grade. We are unaware of any evidence regarding the outcome of Gleason grading based on sections where the presence of basal cells was assessed by immunohistochemistry and there are several precedents for pathology reporting based on morphological rather than immunohistochemical results. For example, prostatic small cell neuroendocrine carcinoma is primarily a morphological diagnosis as such tumours may occasionally be negative for all neuroendocrine markers, which conversely may be expressed by usual prostatic acinar adenocarcinoma.

The Gleason grading system was developed prior to the introduction of immunohistochemistry, and it is recognised that many foci of comedonecrosis pattern of Gleason pattern 5 invasive carcinoma have an at least partially preserved basal layer and would represent IDCP. Recently, Fine et al. demonstrated the presence of a basal cell layer in at least some comedonecrosis pattern 5 in 18 (95%) of 19 cases with 12 (63%) cases showing basal cell marker immunoreactivity in all foci of comedonecrosis [49]. They recommend careful evaluation of the duct/acinar periphery of comedonecrosis foci to detect basal cells, mandatory use of immunohistochemistry in such cases if basal cells are not evident on H&E examination and reconsideration of routine grading of comedonecrosis as pattern 5. However, all these cases were identified in the setting of high-grade high-volume prostate cancer and comedonecrosis IDCP cannot be reliably distinguished from invasive carcinoma by H&E or basal cell immunohistochemistry. Flattened tumour cells and fibroblasts may be morphologically indistinguishable from basal cells while some foci interpreted as invasive carcinoma following immunohistochemistry are likely to represent IDCP in which the patchy basal cells were absent in the immunostained plane of section. In the absence of evidence that the biological outcome of comedonecrosis IDCP is different from that of comedonecrosis invasive prostate cancer, it would be simpler and more reproducible to continue reporting comedonecrosis foci as pattern 5 prostate cancer without resorting to immunohistochemistry.

There is general agreement that IDCP is a risk factor for aggressive cancer, but the presence of IDCP is not included in commonly used prognostic nomograms, which means that there is a danger of the feature being ignored by the urologists. In the report by Khani and Epstein, 11 (18%) of 62 patients with Gleason score 3 + 3 = 6 with IDCP were placed on active surveillance despite the reports noting the association of IDCP with high-grade aggressive disease and 6 (55%) of them subsequently developed disease progression [44]. Moreover, there is no scope for including a text comment on the presence of IDCP when incorporating Gleason scores into databases for research and epidemiological purposes.

Khani and Epstein recommend that IDCP-inv identified in biopsy/TURP specimens should be reported separately as three (19%) of their 16 patients, with IDCP and Gleason score 3 + 3 = 6 as the highest grade on biopsy, had only Gleason score 3 + 3 = 6 cancer in their radical prostatectomy specimens [44]. It should be emphasised, however, that all three of these prostatectomy specimens were only partially submitted for histological examination so the possibility of unsampled high-grade tumour cannot be excluded. Moreover, one of these patients subsequently developed biochemical recurrence while another was stage pT3a on radical prostatectomy, suggesting that at least two of the three patients had clinically significant tumours.

WHO 2016 recommends that IDCP should not be graded, but it is unclear whether this applies to both pure IDCP and IDCP-inv [6]. Since WHO 2016 recommends the use of an in-situ behaviour code (/2) for IDCP, it can be argued that the recommendation is applicable only to pure IDCP. This is an issue that needs to be clarified in future editions of the WHO classification and guidelines.
